# Unraveling the Role of Ubiquitin-Conjugating Enzyme UBE2T in Tumorigenesis: A Comprehensive Review

**DOI:** 10.3390/cells14010015

**Published:** 2024-12-26

**Authors:** Chang Gao, Yan-Jun Liu, Jing Yu, Ran Wang, Jin-Jin Shi, Ru-Yi Chen, Guan-Jun Yang, Jiong Chen

**Affiliations:** State Key Laboratory for Managing Biotic and Chemical Threats to the Quality and Safety of Agro-Products, School of Marine Sciences, Ningbo University, Ningbo 315211, China

**Keywords:** UBE2T, ubiquitin-conjugating enzyme, ubiquitin-proteasome system

## Abstract

Ubiquitin-conjugating enzyme E2 T (UBE2T) is a crucial E2 enzyme in the ubiquitin-proteasome system (UPS), playing a significant role in the ubiquitination of proteins and influencing a wide range of cellular processes, including proliferation, differentiation, apoptosis, invasion, and metabolism. Its overexpression has been implicated in various malignancies, such as lung adenocarcinoma, gastric cancer, pancreatic cancer, liver cancer, and ovarian cancer, where it correlates strongly with disease progression. UBE2T facilitates tumorigenesis and malignant behaviors by mediating essential functions such as DNA repair, apoptosis, cell cycle regulation, and the activation of oncogenic signaling pathways. High levels of UBE2T expression are associated with poor survival outcomes, highlighting its potential as a molecular biomarker for cancer prognosis. Increasing evidence suggests that UBE2T acts as an oncogene and could serve as a promising therapeutic target in cancer treatment. This review aims to provide a detailed overview of UBE2T’s structure, functions, and molecular mechanisms involved in cancer progression as well as recent developments in UBE2T-targeted inhibitors. Such insights may pave the way for novel strategies in cancer diagnosis and treatment, enhancing our understanding of UBE2T’s role in cancer biology and supporting the development of innovative therapeutic approaches.

## 1. Introduction

The ubiquitin-proteasome system (UPS) is a tightly regulated major protein hydrolysis system in the cytoplasm and nucleus of eukaryotic cells, characterized by a multistep cascade of protein modifications leading to the degradation of specific proteins [1]. The key components of the UPS include ubiquitin (Ub), ubiquitin-associated enzymes (UBEs), and the 26S proteasome [2]. This process is initiated by the conjugation of ubiquitin to a protein substrate, which triggers the hydrolysis of the target protein [3]. This conjugation relies on a coordinated interaction between the ubiquitin-activating enzyme (E1), ubiquitin-conjugating enzyme (E2), and ubiquitin ligase (E3) [4]. Briefly, E1 activates ubiquitin by linking the C-terminus of the ubiquitin molecule to a cysteine residue in E1, forming a thioester bond via the energy provided by ATP hydrolysis. Subsequently, a new thioester bond is formed between ubiquitin and E2, transferring ubiquitin to E2. The ubiquitin-carrying E2 enzyme then interacts with E3, transferring ubiquitin to the target substrate, which is ultimately degraded by the 26S proteasome. Studies have demonstrated that the UPS plays a crucial regulatory role in processes such as cell cycle progression, signal transduction, transcriptional regulation, and receptor downregulation [5]. The dysregulation of the UPS is closely associated with numerous diseases, especially cancer, where mutations or the aberrant expression of its components are frequently observed across various cancer types [6]. For instance, in gastric cancer (GC), an imbalance in the UPS disrupts the protein homeostasis network, promoting GC development and progression [7]. Given the central role of the UPS in regulating oncogenic pathways, the development of inhibitors targeting UPS components is regarded as a promising therapeutic strategy, particularly for cancer.

**Figure 1:**
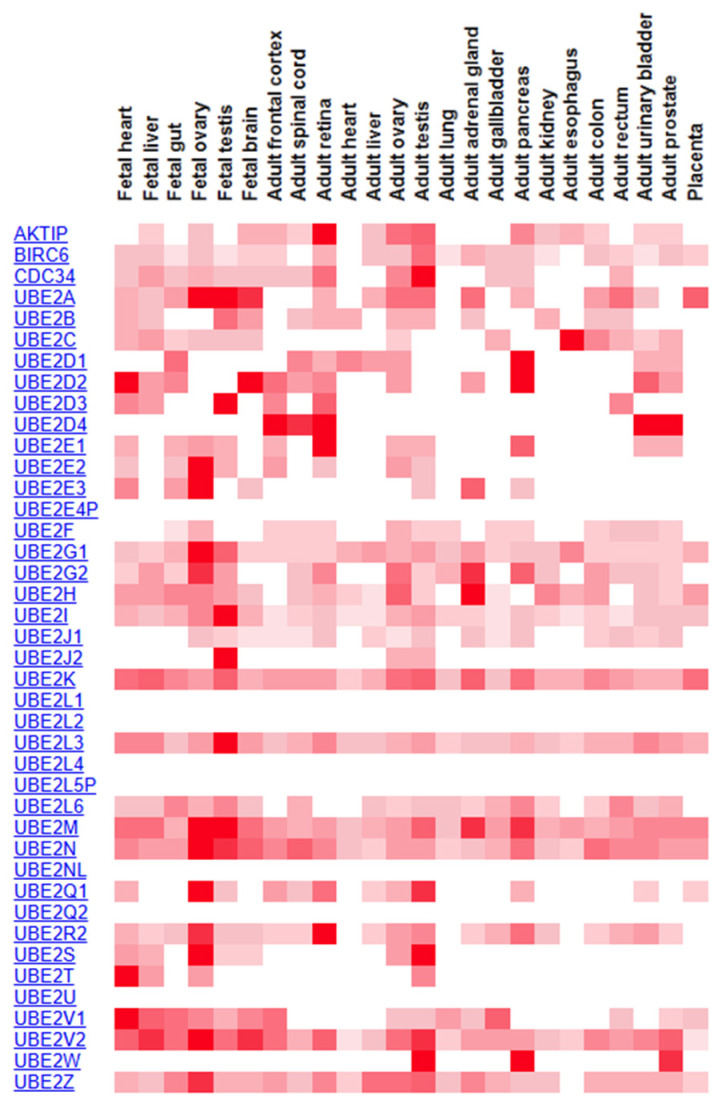
The expression profile of UBE2 across different samples. UBE2T is specifically expressed in the fetal heart, fetal liver, fetal ovary, and adult testis. Based on the total human proteome map (http://www.humanproteomemap.org [18]), accessed on 19 December 2024, obtained by querying the UBE2 family of proteins.

In the UPS, E2 enzymes play a crucial bridging role in transferring ubiquitin molecules to target substrates, serving as central enzymes in the ubiquitin cascade and core components of the system [8]. The UBE2 superfamily includes approximately 40 members, each containing a highly conserved ubiquitin-conjugating (UBC) domain essential for catalytic activity [9]. Using methodologies from existing studies, a human proteomic analysis identified varying expression levels of UBE2 family proteins under physiological homeostasis (Figure 1). The findings revealed that certain UBE2 family genes were highly expressed in the fetal heart, fetal brain, adult ovary, and adult testis, while others, such as UBE2T, UBE2C, and UBE2L3, exhibited high expression across multiple organs [10]. Studies have shown that the dysregulation of UBE2 family proteins in cancer can activate oncogenic signaling pathways. For example, ubiquitin-conjugating enzyme 2C (UBE2C) promotes tumor progression and metastasis through cell cycle regulation [11]. As a key member of the UBE2 family, UBE2T has recently emerged as a significant focus of research regarding its role in cancer development and progression and its underlying mechanisms.

UBE2T is an oncogene overexpressed in various cancers, including Ewing’s sarcoma (ES), pancreatic cancer (PC), glioblastoma (GBM), head and neck squamous cell carcinoma (HNSC), and colorectal cancer (CRC) [12,13,14,15,16]. As an oncogene, UBE2T activates multiple cancer-promoting signaling pathways, including STAT and Wnt [17]. Additionally, UBE2T facilitates tumorigenesis through the ubiquitination and degradation of tumor suppressors such as p53 and BAKC1. Notably, UBE2T expression in different tumors is strongly prognostic, with high UBE2T expression frequently indicating a poor prognosis and reduced patient survival. Given these established findings, UBE2T holds promise as a potential therapeutic target for cancer treatment.

## 2. Structure and Function of UBE2T

**Figure 2:**
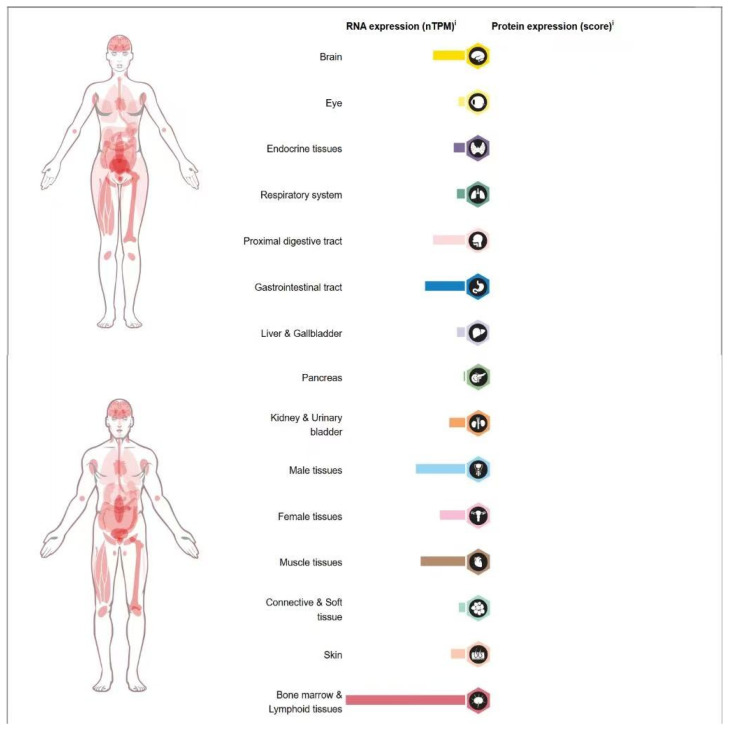
Expression of UBE2T RNA and proteins in various human tissues. Available online at https://www.proteinatlas.org/ENSG00000077152-UBE2T/tissue (accessed on 17 December 2024).

UBE2T, also known as HSPC150 or Fanconi anemia T histone (FANCT), is initially identified as a key E2 enzyme in the Fanconi anemia (FA) pathway [19]. FA is characterized by impaired DNA repair and resulting chromosomal abnormalities [20]. The FA pathway plays a critical role in maintaining genome stability through DNA damage repair, replication fork stabilization, and mitigation of oxidative and mitotic stress [21]. UBE2T contributes to DNA damage repair and alleviates DNA replication stress by ubiquitinating the FANCD2 and FANCI complexes. Notably, UBE2T depletion is also observed in patients with FA. UBE2T is broadly expressed across various tissues and organs in the human body, with the highest expression observed in the bone marrow and lymphatic tissues. Additionally, it exhibits moderate expression in the gastrointestinal tract, muscle tissue, and male tissues, among others (Figure 2). In recent years, high UBE2T expression in various cancers has garnered significant attention. By mediating the ubiquitin-proteasome pathway, UBE2T regulates the degradation and activity of target proteins, influencing several critical processes associated with tumor progression, including cell cycle regulation, signal transduction, and cancer cell stemness. For instance, in malignant melanoma, UBE2T overexpression significantly promotes the proliferation, invasion, and migration of malignant melanoma cells but inhibits apoptosis [22]. In head and neck squamous cell carcinoma (HNSC), UBE2T overexpression also promotes tumor growth by activating NF-κB signaling and inducing ferroptosis [15]. Additionally, UBE2T overexpression in cervical cancer cells enhances self-renewal capacity, indicating its role in promoting cervical cancer stem cell traits [23].

**Figure 3:**
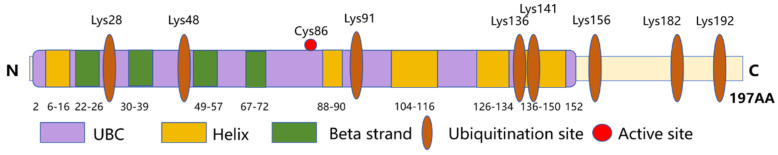
The schematic diagram of the UBE2T domain of the human ubiquitin ligase.

The E2 family of protein structures is highly conserved across various species, featuring a core structure that typically comprises a UBC core domain of 150 amino acids. The human *UBE2T* gene is located on chromosome 1q32.1 and encodes a protein product of 197 amino acids. Similarly, the core structure of UBE2T is a typical UBC structural domain containing several α-helices and β-folds, forming a stable framework to support its catalytic activity (Figure 3). The active site for the reaction of E2 enzyme UBE2T is cysteine 86 (Cys86), located at the center of the UBC structural domain, which forms a thioester bond with activated ubiquitin, the core amino acid for UBE2T’s ubiquitination reaction. Notably, UBE2T itself undergoes ubiquitin modification, containing multiple Lys sites (including Lys28, 48, 91, and 192, among others), and this self-ubiquitination affects protein degradation by regulating UBE2T’s stability. In the FA pathway, FANCL not only acts as an E3 ligase to interact with UBE2T but also regulates UBE2T’s activity by promoting its autoubiquitination, thereby influencing the overall function of the FA pathway. However, studies suggest that UBE2T’s self-ubiquitination may not be entirely dependent on FANCL. It could also be influenced by other E3 ubiquitin ligases or ubiquitin-like proteins, and the precise mechanism remains to be further explored [24]. Lys91 has been shown to affect UBE2T activity through a mono-ubiquitination modification, which is suggested as a negative regulatory mechanism. Specifically, the ubiquitination of Lys91 may inhibit its binding to substrates or E3 ligases, decreasing the catalytic activity of UBE2T, or it may act as a site of ubiquitination, leading to the ubiquitination and degradation of UBE2T itself [25]. In addition to the UBC structural domain, several other regions in the structure of UBE2T undergo conformational changes upon interaction with various E3 ligases, enhancing its binding to specific substrates. For example, UBE2T interacts non-covalently with the ELF structural domain of FANCL via its N-terminus, facilitating the binding between FANCL and ubiquitin. This provides valuable insights into understanding the role of the interaction between UBE2T and FANCL in DNA damage repair [26].

## 3. UBE2T-Mediated Cancer Pathway

**Figure 4:**
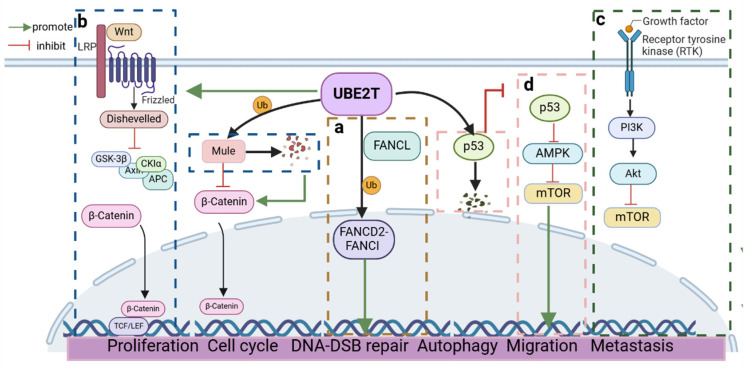
Molecular mechanisms of UBE2T in tumorigenesis. (**a**) FA Pathway, (**b**) Wnt/β-catenin pathway, (**c**) PI3K-AKT pathway, (**d**) p53 signaling pathway.

UBE2T plays a crucial role in regulating biological processes such as cell proliferation, survival, migration, and invasion by interacting with key signaling pathways. Aberrant activation of these pathways is closely linked to the development of various cancers. Therefore, a comprehensive study of UBE2T not only enhances our understanding of cancer pathogenesis but also lays a critical foundation for identifying new therapeutic targets. These insights may lead to innovative strategies for cancer treatment and provide valuable support for clinical interventions (Figure 4).

### 3.1. FA Pathway

FA is a rare, genetically inherited, and phenotypically heterogeneous recessive disorder, characterized by bone marrow failure, developmental abnormalities, and an increased incidence of cancer [27]. This disorder was first described in 1927 by Guido Fanconi, a Swiss pediatrician [19]. The FA pathway is a specialized DNA repair mechanism primarily involved in the repair of interstrand DNA cross-links (ICLs) in the genome, through a series of intricate molecular processes [28]. ICLs are among the most cytotoxic forms of DNA damage, occurring when bases on opposite DNA strands are covalently linked, thereby inhibiting essential processes such as replication and transcription and ultimately impacting cell survival [29]. At least 23 FANC genes encode proteins that are essential for the FA pathway. These proteins work together to form a repair complex that recognizes and repairs ICLs and other DNA damage [30]. Mutations in any of the FA genes result in the development of FA.

UBE2T is a crucial component of the FA pathway, essential for maintaining genomic stability (Figure 4a) [20]. A key step in this pathway is the monoubiquitination of the FA proteins FANCD2 and FANCI, mediated by the multiprotein FA core complex (FA-CC) [31,32]. This process is facilitated by FANCL, an E3 ubiquitin ligase that is part of the FA-CC. The crystal structure of FANCL reveals three domains: an N-terminal E2-like folding (ELF) domain, a central double RWD (DRWD) domain, and a C-terminal RING domain [33]. The RING domain interacts with the E2-E3 interface of UBE2T [26], activating the ubiquitination of FANCD2 and FANCI at specific lysine residues (K561 for FANCD2 and K523 for FANCI) [34]. When DNA undergoes cross-linking damage, FANCD2 and FANCI form a heterodimer, and UBE2T, in conjunction with the FANCL and FA-CC, mono-ubiquitylates the complex [34]. This modified complex is then recruited to the site of ICL damage, where it interacts with a series of repair proteins to form repair complexes that resolve DNA cross-linking damage through mechanisms such as nucleic acid endonuclease cleavage and homologous recombination repair [35]. The ubiquitination of FANCD2 and FANCI is interdependent, with FANCI ubiquitination typically being ubiquitinated after FANCD2, a process that is promoted by FANCD2 [36]. UBE2T deficiency is linked to the FA-T disease subtype, which disrupts the entire FA pathway, leading to DNA damage accumulation, chromosomal instability, and increased apoptosis [37]. Additionally, the activation of the FA pathway is associated with various cancers and their resistance to chemotherapy, with FA patients being particularly susceptible to acute myeloid leukemia (AML) and bone marrow failure [28,38]. Overall, the monoubiquitination of FANCD2 and FANCI by UBE2T and FANCL is central to the activation of the FA pathway.

### 3.2. Wnt/β-Catenin Pathway

The Wnt/β-catenin signaling pathway is a highly conserved pathway comprising several ligands, receptors, co-receptors, intracellular mediators, and transcriptional effectors that regulate critical cellular processes such as cell proliferation, survival, migration, and differentiation [39]. The abnormal activation of the Wnt/β-catenin signaling pathway is strongly linked to various human cancers [40], including hepatocellular carcinoma [41] and cholangiocarcinoma [42]. β-catenin, an important component of the Wnt signaling pathway, plays a critical role in cancer development by modulating several cell events, including proliferation, survival, migration, and stem cell properties [43]. In the inactive state, β-catenin is targeted for degradation by destruction complexes (e.g., Axin, GSK-3β), preventing its nuclear entry. However, upon Wnt signaling activation, these complexes are inhibited, leading to β-catenin accumulation and translocation to nucleus, where it activates cell-survival-promoting genes with the assistance of the transcription factor TCF/LEF. This activation enhances the expression of oncogenes such as c-Myc, survivin, and cyclin D1, contributing to cancer progression and drug resistance. UBE2T levels are often positively correlated with β-catenin in cancer, facilitating Wnt/β-catenin signaling pathway activation by ubiquitinating key proteins (Figure 4b). In hepatocellular carcinoma, UBE2T activates this pathway through several mechanisms, including downregulating Mule (an E3 ubiquitin ligase responsible for β-catenin degradation), promoting β-catenin translocation, activating the MAPK/ERK signaling pathway, and interacting synergistically with other signaling pathways [44,45]. In GBM, UBE2T enhances tumor progression and drug resistance by upregulating survivin and c-Myc via Wnt/β-catenin activation [17]. In non-small cell lung cancer, UBE2T indirectly activates this pathway by ubiquitinating FOXO1, aiding tumor cell survival and contributing to resistance against radiotherapy [46,47]. Additionally, UBE2T promotes β-catenin nuclear translocation through the ubiquitin-mediated degradation of RACK1, facilitating gastric cancer progression [48]. While the association between UBE2T and the Wnt/β-catenin pathway has been explored, specific mechanisms remain unclear, such as UBE2T’s regulation of other β-catenin degradation proteins and its synergistic interactions with Wnt pathway components. A deeper understanding of UBE2T’s role in this pathway could lead to new targeted cancer therapies.

### 3.3. PI3K-AKT Pathway

The PI3K-AKT pathway (phosphatidylinositol 3-kinase/protein kinase B pathway) is a vital cellular signaling cascade that regulates cell growth, proliferation, survival, and metabolism (Figure 4c) [49]. The abnormal activation of this pathway is closely linked to various diseases and cancers, including idiopathic pulmonary fibrosis (IPF) [50], heart failure (HF) [51], and prostate cancer (PCa) [52]. Typically, the pathway is activated by external signals such as growth factors and cytokines, leading to AKT phosphorylation and the regulation of various downstream target proteins that influence numerous cellular functions [53]. UBE2T overexpression has been indicated to enhance the PI3K-AKT pathway activity in multiple cancers, worsening tumor malignancy by increasing the phosphorylation of key components, such as PI3K, Akt, and mTOR, and regulating downstream genes associated with cell survival and anti-apoptosis. However, the specific mTORC pathway primarily influenced by UBE2T remains unclear, and its underlying mechanism requires further investigation. In ovarian cancer, UBE2T may influence the epithelial–mesenchymal transition (EMT) via mTOR targets within the PI3K-AKT pathway [54]. Similarly, in lung adenocarcinoma (LUAD) [55], breast cancer (BCa) [56], renal cell carcinoma (RCC) [57], and osteosarcoma [58], the downregulation of UBE2T has been found to inhibit PI3K/AKT signaling activity and suppress cancer progression. In summary, UBE2T likely enhances PI3K-AKT signaling by modulating key components or promoting the ubiquitination and degradation of specific negative regulators. However, the precise mechanisms underlying this regulation require further investigation. A deeper understanding of UBE2T’s role in the PI3K-AKT pathway could yield novel insights for developing anti-cancer strategies, overcoming drug resistance, and improving cancer immunotherapy efficacy.

### 3.4. p53 Signaling Pathway

The p53 signaling pathway is a fundamental component of cellular homeostasis and tumor suppression [59]. p53, often referred to as the “guardian of the genome”, plays a critical role in cell proliferation, apoptosis, and DNA repair [60]. Under normal physiological conditions, p53 responds to various cellular stress signals including DNA damage, by activating downstream target genes that induce cell cycle arrest, apoptosis, or senescence. Additionally, the cytoplasmic localization of p53 contributes to its tumor suppressor role through the inhibition of AMPK (adenosine monophosphate-activated protein kinase) activity, which subsequently suppresses autophagy [61]. Recent research has highlighted the interaction between UBE2T and the p53 signaling pathway in the context of cancer. UBE2T is known to promote tumor cell proliferation and survival by ubiquitinating and degrading p53 through the proteasome pathway, thereby compromising its tumor-suppressive abilities (Figure 4d). This mechanism has been validated in pancreatic [62], lung adenocarcinomas [63], hepatocellular carcinomas [64], and colorectal carcinomas [65]. Specifically, in pancreatic cancer, the E3 ubiquitin-conjugating enzyme RING1 has been identified as a key player that interacts with UBE2T to mediate the ubiquitination and degradation of p53 [62]. The regulation of p53 by UBE2T is complex. Beyond direct degradation, UBE2T influences p53’s functionality by affecting its subcellular localization. In the nucleus, p53 acts as a transcription factor that activates autophagy-related genes, promoting autophagy. Conversely, in the cytoplasm, p53 may inhibit autophagy through an undefined mechanism. Notably, in non-small cell lung cancer (NSCLC), UBE2T overexpression has been shown to reduce the accumulation of p53 in the cytoplasm, which activates the AMPK/mTOR signaling pathway and enhances autophagy in tumor cells. This interplay is crucial for tumor progression and drug resistance [66]. In conclusion, UBE2T significantly impacts the p53 signaling pathway, influencing the onset and progression of various cancers. Understanding the intricate mechanisms by which UBE2T regulates p53 could unveil new therapeutic targets and strategies for cancer treatment, particularly in overcoming drug resistance and enhancing the effectiveness of cancer therapies. Further research into the UBE2T–p53 axis is essential for developing novel interventions aimed at restoring p53 function and inhibiting tumor growth.

## 4. Role of UBE2T in Cancer Development

**Figure 5:**
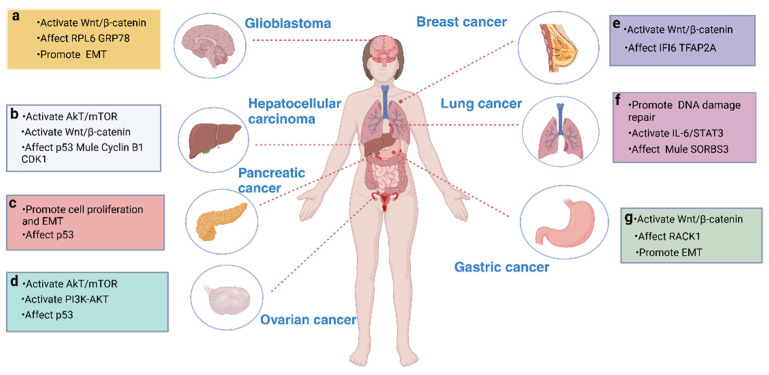
The role of UBE2T in various tumorigenesis.

There has been some progress on targeted therapy for cancer, for example, in gastrointestinal tumors [67]. A series of studies have shown that UBE2T is overexpressed as an oncogene in various types of cancer, including hepatocellular carcinoma, lung adenocarcinoma, pancreatic cancer, breast cancer, ovarian cancer, gastric cancer, and glioblastoma (Figure 5). Through its interactions with E3 ligases and other proteins, UBE2T plays a key role in several fundamental processes of cancer progression, including cell proliferation, invasion, metastasis, and cancer stem cell maintenance, and has also been linked to drug resistance in certain cancers (Table 1). These findings suggest that UBE2T may serve as a potential therapeutic target for many cancers, providing new avenues for the development of drugs targeting cancer therapy.

### 4.1. Glioblastoma

GBM is the most aggressive primary brain tumor in adults, characterized by a high recurrence rate and low survival rate, making it particularly challenging to treat [72]. Currently, temozolomide (TMZ) chemotherapy is a common clinical treatment; however, patient prognosis remains poor due to the rapid development of drug resistance [73,74]. In this context, UBE2T plays a critical role in the invasiveness, migratory capacity, and drug resistance mechanisms of GBM. Ribosomal proteins are not only essential for protein biosynthesis but also play vital roles in DNA replication and repair, RNA splicing and modification, and transcription—processes crucial for cell proliferation, metastasis, and apoptosis. RPL6, a member of the ribosomal protein family, has been shown to inhibit cell proliferation and promote apoptosis. In GBM, UBE2T ubiquitinates and degrades RPL6 in an E3 ligase-independent manner, thereby promoting tumor progression [14]. Additionally, GRP78, a regulator known to enhance tumorigenesis, metastasis, and drug resistance, has been identified as a key target of UBE2T [68]. Recent studies indicate that UBE2T promotes epithelial–mesenchymal transition (EMT) by stabilizing GRP78 through Lys63-linked ubiquitination, significantly enhancing GBM invasion and migration. Furthermore, UBE2T is a crucial regulator of GBM chemoresistance, promoting TMZ resistance by activating the Wnt/β-catenin signaling pathway [17]. Using a mouse xenograft model, Yang et al. demonstrated that UBE2T overexpression induces Wnt/β-catenin signaling activation, thereby promoting tumor progression and TMZ resistance (Figure 5a). Notably, the combination of TMZ with UBE2T inhibitors resulted in significantly greater tumor growth inhibition in experimental settings. These findings suggest that targeting UBE2T may offer an effective strategy to overcome TMZ resistance and inhibit the malignant progression of GBM.

### 4.2. Hepatocellular Carcinoma

HCC is the most common form of liver cancer and one of the most prevalent malignancies threatening human health [75]. The development of HCC is a multistep, progressive process characterized by dysregulation of various tumor suppressor genes and oncogenes [76,77]. The complex molecular mechanisms underlying its onset and progression remain poorly understood. UBE2T has been identified as a key gene in HCC [78,79,80]. Elevated UBE2T expression correlates closely with HCC occurrence, progression, and poor patient prognosis. In HCC, UBE2T exerts oncogenic effects by activating multiple signaling pathways, including AKT/mTOR [81] and Wnt/β-catenin [45], which promote cell proliferation, invasion, migration, and stemness. For example, UBE2T enhances HCC progression by promoting K63-linked ubiquitination of Akt, which subsequently activates the Akt/β-catenin signaling pathway. Recent studies have also shown that SUMOylation, a reversible post-translational modification, regulates protein activity and stability, influencing HCC cell proliferation and migration [82]. To be specific, UBE2T acts as a conjugase enzyme that can also be SUMOylated. SUMO-specific protease 1 (SENP1) activates signaling pathways such as Akt by de-SUMOylating UBE2T, thereby modulating its function. Additionally, UBE2T activates the Wnt/β-catenin signaling pathway by affecting the localization and activation of β-catenin transcription. The E3 ligase Mule interacts directly with UBE2T during ubiquitination [45]. Studies have indicated that Mule regulates Wnt signaling in colorectal cancer by degrading β-catenin [83]. Interestingly, in HCC, UBE2T may directly ubiquitinate and degrade Mule rather than merely acting solely as a mediator for transferring ubiquitin to the E3 ligase. In terms of radiation resistance, UBE2T can monoubiquitinate H2AX/γH2AX-related proteins, promoting the DNA damage response and G2/M cell cycle arrest following ionizing radiation, thus conferring radioresistance to HCC [84]. Furthermore, UBE2T accelerates cancer progression by promoting the degradation of tumor suppressors through ubiquitination. Uncontrolled cell growth is a hallmark of cancer cells, and UBE2T upregulation promotes tumor cell growth by accelerating the ubiquitin-mediated degradation of p53 proteins [64]. This effect may be related to multiple p53 downstream signaling pathways involved in regulating G1/S and G2/M cell cycle checkpoint (Figure 5b). Although UBE2T mediates p53 ubiquitination, the specific E3 ligase that binds to it remains unclear. Moreover, further studies have revealed that inhibiting UBE2T expression downregulates cyclin B1 and cyclin-dependent kinase 1 (CDK1) levels, leading to G2/M cell cycle arrest and the inhibition of HCC cell proliferation [85]. In conclusion, UBE2T’s significance in HCC lies in its role as a multifunctional oncogene that drives cancer progression, treatment resistance, and poor prognosis. Understanding the mechanisms by which UBE2T promotes HCC development may guide the creation of novel therapeutic strategies targeting UBE2T, potentially improving outcomes for HCC patients.

### 4.3. Pancreatic Cancer

Pancreatic cancer (PC) is a highly fatal malignant tumor of the digestive system, with rising morbidity and mortality rates worldwide [86]. Due to its subtle early symptoms, rapid progression, and low chemotherapy efficacy of chemotherapy, the prognosis for PC patients remains extremely poor, with a 5-year overall survival rate of only 10%. Currently, gemcitabine is the first-line drug for PC treatment; however, widespread drug resistance significantly challenges clinical outcomes and threatens long-term patient survival [87]. Research has shown that pyrimidine metabolism and replication stress, which are closely interconnected, play crucial roles in gemcitabine resistance. Jiang et al. demonstrated that UBE2T levels are positively correlated with clinical resistance to gemcitabine. UBE2T alleviates transcriptional repression of ribonucleotide reductase subunits M1 (RRM1) and M2 (RRM2), thereby reducing replication stress and promoting pyrimidine biosynthesis. This is achieved by ubiquitinating and inducing the degradation of p53 at the K291 and K292 residues through its interaction with the E3 ubiquitin ligase RING155, which further contributes to gemcitabine resistance [62]. This process promotes pyrimidine biosynthesis, thus conferring gemcitabine resistance. Additionally, high UBE2T expression promotes PC cell proliferation and invasion, enhancing the cells’ invasive capabilities by facilitating EMT (Figure 5c) [13]. These findings suggest that UBE2T not only plays a critical role in the progression of PC but can also serves as a biomarker for predicting disease progression, prognosis, and therapeutic response. Targeting UBE2T presents a promising strategy to enhance the efficacy of gemcitabine, providing a compelling scientific basis for developing novel therapeutic approaches aimed at UBE2T inhibition..

### 4.4. Ovarian Cancer

Ovarian cancer (OC) is the deadliest gynecologic malignancy, leading in both morbidity and mortality among gynecologic cancers, with a 5-year overall survival rate of only 41% [88]. The current first-line treatment includes cytoreductive surgery and platinum-based chemotherapy; however, most OC patients develop cisplatin (DDP) resistance, significantly contributing to a poor prognosis [89]. Therefore, identifying novel diagnostic and prognostic biomarkers as well as therapeutic targets is essential for improving treatment outcomes. UBE2T mRNA and protein levels are upregulated in OC tissues and cells, correlating with advanced disease stages and a poor prognosis [90]. It is known that p53 aggregation can reverse DDP chemoresistance in OC [69], and high UBE2T expression accelerates the ubiquitin-mediated degradation of p53, increasing cellular resistance to platinum-based drugs (Figure 5d). Notably, Zhu et al. identified a circular RNA (circRNA), circNUP50, that enhances platinum resistance by promoting the ubiquitination and degradation of p53 through direct binding with both p53 and UBE2T [69]. This finding suggests that certain circRNAs are critical molecular targets regulating the p53 ubiquitination pathway, and antagonizing circNUP50 expression may offer a therapeutic strategy to overcome DDP resistance. Additionally, Huang et al. demonstrated that knocking down UBE2T significantly inhibits the growth, proliferation, and invasion of OC cells, as evidenced by reduced cell viability, fewer colonies, and decreased invasiveness. Conversely, UBE2T upregulation promotes malignant progression in OC. Specifically, increased UBE2T expression inhibits autophagy and promotes epithelial–mesenchymal transition (EMT) by activating the AKT/mTOR axis, facilitating cell proliferation and invasion. The PI3K-AKT pathway has also been implicated in regulating UBE2T’s effects on OC cell proliferation and invasion, indicating that UBE2T may influence the oncogenic role of EMT in OC through this pathway [91]. In summary, UBE2T plays a crucial role in the initiation, progression, and metastasis of OC. An in-depth investigation into the mechanisms of UBE2T will provide novel insights and potential targets for OC diagnosis and treatment.

### 4.5. Breast Cancer

Breast cancer is one of the most prevalent malignancies in women and the second leading cause of cancer-related death after lung cancer [92]. Triple-negative breast cancer (TNBC) is one of the most aggressive subtypes of breast cancer [93]. UBE2T is recognized as an oncogene in breast cancer, with studies linking high UBE2T expression to tumorigenesis, progression, and poor prognosis. Qiao et al. demonstrated the significant tumor-promoting effect of UBE2T in vivo using a mouse xenograft model [94], highlighting its regulatory role in the cell cycle and oncogenic signaling pathways [95]. Specifically, UBE2T overexpression resulted in the upregulation of the mTORC1 and TNFA signaling pathways, which facilitated cell cycle progression and inhibited apoptosis, thereby promoting breast cancer cell growth. Additionally, UBE2T mitigated DNA replication stress and blocked apoptotic pathways by upregulating IFI6 expression, enhancing cell proliferation and survival. IFI6, an interferon-stimulated gene overexpressed in various cancers, stabilizes mitochondrial function, contributing to apoptosis inhibition and tumor growth [96]. Moreover, the transcription factor AP-2α (TFAP2A) was identified as essential for UBE2T transcriptional upregulation, underscoring the complexity of this regulatory network. While IFI6 overexpression can reverse the DNA replication stress and growth inhibition caused by UBE2T knockdown, it may also promote breast cancer cell growth through non-UBE2T-dependent pathways, influencing the multifaceted tumor phenotype (Figure 5e). These findings deepen our understanding of breast cancer pathogenesis and offer novel insights for developing multi-targeted and precise therapeutic strategies.

### 4.6. Lung Cancer

Lung cancer is one of the most common malignant tumors worldwide and is generally divided into two main types: non-small cell lung cancer (NSCLC) and small cell lung cancer (SCLC). NSCLC is the most prevalent, accounting for approximately 85% of lung cancer cases, and includes subtypes such as lung adenocarcinoma (LUAD), squamous cell carcinoma, and large cell carcinoma [97]. The dysregulation of UBE2T in lung cancer has been shown to initiate oncogenic signaling pathways, leading to tumor progression and metastasis [70]. In LUAD, UBE2T expression is upregulated, and this increased expression correlates with larger tumor size, lymph node metastasis, distant metastasis, and poor prognosis. UBE2T promotes LUAD cell proliferation by regulating various signaling pathways. The IL-6/STAT3 signaling pathway, commonly activated in cancer, involves IL-6 binding to IL-6R, which activates receptor-associated JAK, leading to the phosphorylation of STAT3. Phosphorylated STAT3 then translocates to the nucleus, promoting the transcription of genes related to cancer progression. UBE2T also activates the p53/AMPK/mTOR and Wnt/β-catenin signaling pathways [47]. Additionally, UBE2T exerts oncogenic effects in LUAD by mediating the ubiquitin-mediated degradation of specific proteins. For instance, UBE2T negatively regulates RORA by promoting the ubiquitination and degradation of the intermediate transcription factor Pre-B cell leukemia transcription factor 1 (PBX1), thereby advancing LUAD progression. RORA, a member of the retinoid-related orphan receptor (ROR) family, has been shown to inhibit cell proliferation and migration in LUAD cell lines [98]. Furthermore, Jiang et al. found that SORBS3, a tumor suppressor protein, is significantly downregulated in LUAD tissues and cells due to UBE2T-mediated ubiquitination and the degradation of SORBS3, which activates IL-6/STAT3 signaling in LUAD cells, thus promoting tumor growth [71]. Notably, UBE2T has also been associated with immune evasion of LUAD tumor cells, which may become a focus of future studies (Figure 5f). High levels of FANCI expression have been observed in NSCLC tumor tissues, and FANCI knockdown has been shown to inhibit cell proliferation, migration, invasion, cell cycle progression, and EMT in vitro and ex vivo [99]. FANCI, a member of the FA protein family, regulates the cell cycle during the S and G2 phases, thereby maintaining DNA stability. UBE2T promotes the monoubiquitination of FANCI by interacting with the FA core complex FANCL, enhancing DNA double-strand break repair and supporting NSCLC development. Collectively, these findings underscore the significant role of UBE2T in LUAD progression, suggesting that its identification as a core player in NSCLC provides a foundation for future research and the development of targeted therapies to improve the prognosis of NSCLC patients.

### 4.7. Gastric Cancer

Gastric cancer is one of the most prevalent cancers worldwide, with UBE2T reported to be significantly upregulated in gastric cancer tissues [100]. High expression levels of UBE2T are associated with poor differentiation, advanced tumor classification (T classification), and an overall poor prognosis. UBE2T has been shown to activate the Wnt/β-catenin signaling pathway, which is critical in cancer progression [48]. By mediating the ubiquitination and degradation of the scaffolding protein RACK1, UBE2T hyperactivates the Wnt/β-catenin pathway, thereby promoting tumor growth. Furthermore, UBE2T-mediated degradation impacts other signaling proteins essential for cell cycle progression and epithelial–mesenchymal transition (EMT), further enhancing the invasiveness of gastric cancer (Figure 5g). Li et al. found that in gastric cancer, UBE2T is positively correlated with E2F5, which upregulates UBE2T and contributes to the malignancy and poor prognosis associated with the disease [101]. This relationship suggests a potential E2F5/UBE2T axis in gastric cancer, opening new avenues for the diagnosis and treatment of this malignancy. Collectively, the available evidence supports UBE2T as a promising biomarker and therapeutic target in gastric cancer.

### 4.8. Multiple Myeloma

Multiple myeloma (MM) is a malignancy of plasma cells and ranks as the second most common hematologic tumor [102]. Research has demonstrated that UBE2T is highly expressed in multiple myeloma cell lines and patient samples, with its expression closely linked to disease progression and aggressiveness [103]. The UBE2T gene is located in the 1q32 region, where 1q32 amplification (1q gain/amp) has been identified as a poor prognostic marker in MM patients. Notably, Li et al. reported a significant association between UBE2T overexpression and poor patient outcomes, particularly in MM cells with 1q gain/amplification [104]. Furthermore, UBE2T plays a pivotal role as a key regulator of homologous recombination (HR) repair, and its overexpression has been shown to reduce chemotherapy sensitivity in MM cells [105]. Given these findings, UBE2T represents a promising therapeutic target. Targeting UBE2T could offer novel strategies to overcome chemotherapy resistance, reduce genomic instability, and improve treatment outcomes for high-risk MM patients.

### 4.9. Others

In addition to the previously discussed tumor types, UBE2T plays a significant role in various other cancers. In esophageal squamous cell carcinoma (ESCC), UBE2T mediates the upregulation of several signaling pathways, including the p53 and FA pathways, and may serve as a prognostic marker for ESCC [106]. In retinoblastoma, high UBE2T expression enhances Th2 cell immune infiltration by activating the STAT3 pathway, promoting tumor formation and progression; however, further investigation into the specific UBE2T proteins involved in this activation is warranted [107,108]. Additionally, elevated UBE2T levels are closely associated with patient prognosis, highlighting the necessity for ongoing research and the development of related therapeutic strategies. In intrahepatic cholangiocarcinoma (ICC), UBE2T serves as an independent indicator of poor prognosis and a potential drug target for future molecular-targeted therapies [109]. In endometrial cancer, increased UBE2T expression may inhibit antitumor immune responses and is implicated in tumorigenesis, metastasis, and invasion, correlating with poorer survival outcomes. Wang et al. found a significant correlation between mitochondrial proteins and UBE2T expression [110], suggesting that overexpressed mitochondrial genes promote tumor cell proliferation and metastasis through their roles in energy metabolism, redox regulation, and apoptosis [111]. Thus, UBE2T may facilitate endometrial cancer development by upregulating mitochondrial protein expression. Furthermore, high UBE2T levels reduce immune cell infiltration, such as T and B cells, impairing the immune system’s ability to recognize and eliminate tumor cells, thereby inhibiting the body’s antitumor immune response.

## 5. UBE2T Inhibitors and Related Regulatory Factors

**Figure 6:**
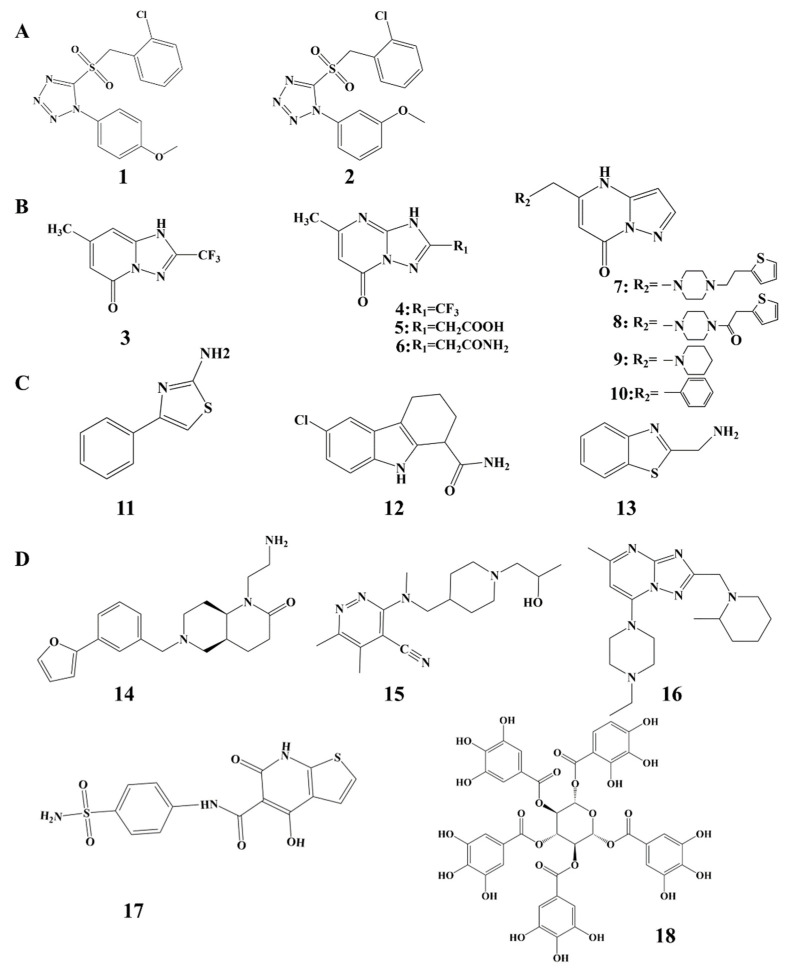
Chemical structures of UBE2T inhibitors. (**A**) Sulfone-thiazole compounds, (**B**) pyrimidinone core compounds and their structural analogs, (**C**) heterocyclic aromatic compounds, and (**D**) other small molecule compounds.

The overexpression of UBE2T in various cancers and its involvement in critical processes such as cell proliferation, invasion, metastasis, and drug resistance make it an attractive target for cancer drug development. However, the crystal structure of UBE2T reveals a lack of suitable small-molecule binding sites, contributing to low drug success rates. While several UBE2T inhibitors have been identified through various methods, effective inhibitors for clinical use remain elusive. Therefore, continued efforts are essential to develop more potent UBE2T inhibitors. Additionally, microRNAs have been shown to target UBE2T across different cancers, inhibiting its oncogenic effects and offering another potential strategy for therapeutic intervention (Figure 6).

### 5.1. Sulfone-Thiazole Compounds

Anantharajan et al. identified two compounds with significant inhibitory activity against UBE2T: **2** (IC_50_ = 0.16 μM) and **9** (IC_50_ = 0.57 μM) (Figure 6A), with **2** demonstrating a stronger inhibitory effect [112]. Both compounds feature a sulfonyl tetrazolium core, which covalently binds to UBE2T’s Cys86, effectively blocking the ubiquitin transfer reaction. A crystal structure analysis revealed that these inhibitors interact with key residues within UBE2T’s catalytic pocket, particularly I74, H76, and N78, near Cys86. Their inhibitory effects are not solely due to covalent binding; additional interactions, including water molecule bridging and hydrogen bonds facilitated by the nitrogen atoms in the tetrazole ring, contribute to the stability of the inhibitors within the binding pocket. While **2** and **1** exhibited weak inhibition of other E2 enzymes, such as UBE2K, their high selectivity for UBE2T can be attributed to the unique structural features of UBE2T, which create an ideal binding site for the sulfotetrazole ring. Although covalent binding can lead to non-specific toxicity, the specific interaction of these compounds with UBE2T minimizes this risk [113]. Future optimization efforts should focus on the tetrazole sulfone skeleton, with rational modifications to enhance selectivity and stability, thereby improving the clinical potential of UBE2T inhibitors.

### 5.2. Pyrimidinone Core Compounds and Their Structural Analogs

Through further research, Loh et al. utilized 19F-NMR fragment screening to identify fragments capable of binding UBE2T, determined their binding sites via X-ray crystallography, and identified two new binding pockets on UBE2T [114]. One pocket, located near Cys86 (the active site of UBE2T), may interfere with ubiquitin transport, potentially inhibiting enzyme activity. The second pocket, distant from the catalytically active site but near the interaction interface between UBE2T and the FANCL RING, suggests that compounds binding here could disrupt UBE2T’s interaction with its substrate or chaperone E3 ligase. The co-crystal structure of **3** (IC_50_ > 900 μM) with UBE2T revealed binding to the region between α1 and α2, which forms the protein–protein interaction (PPI) interface between UBE2T and E3. This finding suggests that PPI libraries could serve as a resource for discovering novel UBE2T inhibitors. Further optimization of the **3** fragment was conducted to develop more effective inhibitors. To enhance synthetic feasibility, a nitrogen atom was introduced, shifting the core structure from pyridone to pyrimidone, resulting in **4**. Modifications, including the addition of acid and amide groups (**5** and **6**, respectively), were also explored, but no significant activity improvements were observed. Notably, a compound (**7**, IC_50_ = 288.2 μM) with an approximately 10-fold increase in binding affinity and a 3-fold increase in potency was identified. Experimental evidence further confirmed its inhibitory effect on UBE2T enzyme activity. Structural analogs, such as **8** (IC_50_ = 427.4 μM), **9** (IC_50_ = 332.2 μM), and **10** (IC_50_ = 621.6 μM), exhibited measurable IC_50_ values (Figure 6B). Although these fragments currently lack significant inhibitory effects, they may serve as preliminary inhibitors or potential lead compounds, providing a foundation for developing more potent UBE2T inhibitors.

### 5.3. Heterocyclic Aromatic Compounds

Morreale et al. conducted fragment screening using differential scanning fluorimetry (DSF), bio-layer interferometry (BLI), and one-dimensional nuclear magnetic resonance (NMR), confirming through in vitro ubiquitination assays that fragments **11**, **12**, and **13** (Figure 6C) bind to a novel conformational pocket in UBE2T, exhibiting inhibitory effects [115]. An NMR chemical shift perturbation analysis and X-ray crystallography identified a metastable pocket near Cys86, situated on the loop between the β4 strand and 310 helixes within the E2 fold. A notable feature of these fragments is the presence of an amino group (-NH_2_), which acts as a hydrogen-bond donor, stabilizing interactions within UBE2T’s pocket. Other polar groups in the fragments also contribute as hydrogen bond donors or acceptors, enhancing interactions with polar and hydrophobic residues on UBE2T. Specifically, **13**’s benzothiazole structure allows the sulfur atom to form a hydrogen bond with the NH backbone of Ile74, further stabilizing the **13**-UBE2T complex. In vitro experiments demonstrated that **11**, **12**, and **13** effectively inhibited FANCD2 ubiquitination by UBE2T in a concentration-dependent manner (e.g., at 2.5 mM) while showing lower inhibitory effects on other E2 enzymes like UbcH5c, indicating selective affinity for UBE2T. A mutational analysis revealed that the P73K mutation in UBE2T significantly reduced the inhibitory effects of **13** and **11**, while **12** maintained its activity. The UBE2T P73K variant also exhibited decreased activity compared to the wild type, highlighting the importance of this site for UBE2T function. This information suggests that structure–activity relationship (SAR) optimization could focus on enhancing interactions at P73 and its surroundings to improve the effectiveness of **1** and **3** as UBE2T inhibitors. In summary, the inhibitory effects of **11**, **12**, and **13** are closely linked to their binding modes, which facilitate UBE2T inhibition through hydrogen bonding and hydrophobic interactions within the metastable pocket. Their low molecular weights align with fragment-based design criteria, and further optimization could enhance the activity and selectivity of these inhibitors, laying a strong foundation for developing effective UBE2T inhibitors.

### 5.4. Other Small Molecule Compounds

In addition to the aforementioned inhibitors, other compounds targeting UBE2T, which is implicated in various signaling pathways and cancers, have been reported. For instance, **14**, **15**, and **16** inhibit the UBE2T/FANCL-mediated ubiquitination of FANCD2 in the FA pathway, while **17** targets gastric cancer and **18** targets pancreatic cancer (Figure 6D) [29,48,116].

A crucial step in the activation of the FA pathway is the mono-ubiquitination of the FANCD2-FANCI complex mediated by UBE2T- and FANCL. This pathway’s activation is associated with chemoresistance in various cancers, making it an attractive therapeutic target. However, developing drugs that target protein–protein interactions is challenging, and few selective inhibitors of the ubiquitin-coupled pathway have been identified [117]. Cornwell et al. discovered several small-molecule compounds, including **14** (IC_50_ = 56.0 μM), **17** (IC_50_ = 45.4 μM), and **16** (IC_50_ = 14.8 μM), through a high-throughput-screening (HTS)-compatible assay [29]. Importantly, in vitro and cellular experiments indicated that these compounds do not act as traditional inhibitors of UBE2T; instead, they inhibit the FA pathway by disrupting the ubiquitination reaction mediated by UBE2T and FANCL. Therefore, **14**, **15**, and **16** should be viewed as inhibitors of the UBE2T/FANCL-mediated FANCD2 ubiquitination reaction rather than direct UBE2T inhibitors. Among these, **16** shows the most promise due to its low IC_50_ value, minimal cytotoxicity, and significant inhibitory effect, positioning it as a potential FA pathway inhibitor that could enhance cancer therapy effectiveness. In contrast, **14** and **15** have higher IC_50_ values, making them less effective, and **14** exhibits some cytotoxicity at elevated concentrations, limiting its utility in cellular experiments.

UBE2T is crucial in promoting gastric cancer progression by mediating the ubiquitination and degradation of RACK1, which leads to the over-activation of the Wnt/β-catenin signaling pathway—a key driver of tumor progression. Researchers screened the Chemdiv and SPECS small-molecule compound libraries and identified a novel UBE2T inhibitor **17**. This compound specifically targets the catalytic site Cys86, effectively blocking UBE2T-mediated Wnt/β-catenin over-activation and thus inhibiting GC progression [48]. Molecular dynamics simulations revealed that compound **17** fits into UBE2T’s active pocket, forming hydrogen bonds that inhibit RACK1 ubiquitination. This action reduces the nuclear translocation of β-catenin, consequently restraining Wnt/β-catenin pathway activation. The binding affinity of **17** to UBE2T was measured with a K_D_ value of 50.5 μM. Additionally, the IC_50_ values of **17** in gastric cancer cell lines were 11.88 μM for HGC27 and 6.93 μM for MKN45, indicating selective inhibitory effects on cancer cell growth compared to normal gastric mucosal cells (GES-1: 16.8 μM). Importantly, **17** demonstrates lower toxicity toward normal cells compared to existing Wnt/β-catenin inhibitors used in other cancers, positioning it as a promising targeted therapeutic candidate for gastric cancer. This represents a novel therapeutic approach for patients, particularly those with aberrant Wnt/β-catenin pathway activation.

1,2,3,4,6-O-Pentagalloylglucose **18** is a naturally occurring galloyl-β-d-glucose compound that has emerged as a promising inhibitor of UBE2T, particularly in the context of pancreatic cancer treatment. Research has shown that **18** exhibits significant synergistic effects when used in combination with gemcitabine, a standard chemotherapy agent for pancreatic cancer [118]. 1,2,3,4,6-O-Pentagalloylglucose **18**’s tumor-suppressive activity is attributed to its multiple galloyl groups, which contain hydroxyl groups that specifically bind to the active site of UBE2T, notably at cysteine residue Cys86, with a dissociation constant (K_D_) of 23.94 nmol/L [116]. This binding effectively inhibits the enzymatic activity of UBE2T, leading to a reduction in the ubiquitination of p53. The resultant decrease in p53 degradation prevents the overexpression of ribonucleotide reductase subunits RRM1 and RRM2, which are implicated in gemcitabine resistance in pancreatic cancer cells. In both in vivo and in vitro studies, the combination of **18** with gemcitabine has demonstrated a significant reduction in the half-maximal inhibitory concentration (IC_50_) of gemcitabine, indicating that **18** enhances the sensitivity of pancreatic cancer cells to this chemotherapeutic agent. Furthermore, this combination therapy has been shown to significantly inhibit tumor growth and prolong survival in animal models of pancreatic cancer. Importantly, compound **18** has exhibited low toxicity, highlighting its potential for clinical application as a sensitizer in cancer therapy. However, the pathway to the clinical translation of new therapeutic agents is complex and requires several steps. Future efforts will need to focus on optimizing the chemical structure of **18** as well as conducting detailed pharmacokinetic and toxicological studies to ensure the safety and efficacy of this novel combination therapy in clinical settings.

### 5.5. MicroRNA

MicroRNAs (miRNAs) are small non-coding RNAs that serve as valuable biomarkers for diagnosing and prognosing diseases, particularly cancers [119,120]. Numerous studies have demonstrated that miRNAs inhibit cancer cell proliferation, invasion, migration, and EMT by negatively regulating UBE2T across various cancers. For example, miR-1322 and miR-1305 are key regulators in HCC, targeting UBE2T to impede cancer development and progression [121,122]. Similarly, miR-490-5p downregulates UBE2T, enhancing anticancer effects and reducing paclitaxel (PTX) resistance in NSCLC [123]. In malignant melanoma, miR-498 inhibits progression by targeting UBE2T [22], while in clear cell renal cell carcinoma (ccRCC), miR-182-5p suppresses UBE2T protein expression, thereby inhibiting cell proliferation, migration, and invasion by targeting UBE2T mRNA [124]. These studies highlight the role of miRNAs in cancer suppression through UBE2T targeting and suggest their potential as therapeutic targets. Future development of agents that regulate miRNAs could lead to novel therapeutic strategies, offering more effective and personalized treatments for patients with UBE2T-overexpressing cancers.

## 6. Therapeutic Potential and Future Directions

The dysregulation of ubiquitination is closely associated with cancer progression, with E2 ubiquitin-conjugating enzymes playing critical roles in various biological processes linked to tumorigenesis [125]. Targeting the E2 enzyme family, particularly UBETT, presents a promising approach for cancer therapy. UBE2T is recognized as an oncogene and is associated with multiple cancer types, which make the development of UBE2T-targeted modulators an attractive focus for drug development. However, the specific role of UBE2T as a therapeutic target in certain cancers remains largely unexplored. Further investigation is needed to delineate UBE2T’s mechanisms and its roles in various cell signaling pathways, which could reveal new directions for cancer treatment. With advancements in genomics, proteomics, metabolomics, and bioinformatics, the regulatory network of UBE2T is expected to be further elucidated. As a target for next-generation cancer diagnostics and therapeutics, UBE2T holds a promise for innovative strategies in tumor diagnosis and treatment.

Recent advances in genome-wide association studies have identified UBE2T as a predicted target of multiple miRNAs implicated in cancer progression. While several miRNAs regulating UBE2T have been discovered, the complete regulatory network remains unclear. Future research should integrate multi-omics data to construct a comprehensive model of the UBE2T-miRNA interaction, elucidating its mechanisms across different cancer types and identifying additional key regulators. Furthermore, UBE2T expression levels, influenced by the tumor microenvironment, correlate with immunoregulatory processes, including tumor-associated immune cell infiltration. This correlation may arise from variations in tumor cell types and extracellular conditions, highlighting the need for ongoing investigation into these mechanisms, which will be crucial for future UBE2T research.

Recent research on UBE2T inhibitors has advanced significantly, demonstrating promising therapeutic potential when combined with treatments such as radiotherapy, chemotherapy, and immunotherapy. This combination approach is expected to enhance treatment efficacy and improve survival outcomes for cancer patients. Future studies should focus on optimizing the chemical properties of UBE2T inhibitors to minimize toxicity and maximize effectiveness across various cancer types. Additionally, exploring multi-targeted combination strategies involving UBE2T inhibitors with existing therapies could lead to more comprehensive tumor suppression. Encouragingly, although existing direct drug inhibitors of UBE2T lack specificity, limiting its therapeutic potential, the negative self-regulation mechanism of UBE2T offers a promising new approach. By targeting proteins involved in its self-ubiquitination process, it may be possible to develop more specific regulators, thereby enhancing the effectiveness of targeted therapies.

Despite the potential of UBE2T as a target in cancer therapy, significant challenges remain. First, functional analyses reveal that UBE2T acts as an oncogene that promotes tumorigenesis and progression, with its effects varying across different tumor types and cellular environments. This variability, likely influenced by the complex tumor microenvironment, complicates the design of effective therapeutic strategies targeting UBE2T. Second, the intricate mechanisms underlying UBE2T’s function pose another challenge. UBE2T is known to interact with multiple signaling pathways and various molecules, yet the specific mechanisms and associated E3 enzymes are still not fully understood [126]. As a result, targeting UBE2T may inadvertently affect other signaling pathways, increasing the risk and complexity of treatment approaches.

In conclusion, UBE2T holds significant promise as a targetable oncogene for cancer diagnosis, treatment, and prognosis. To fully harness this potential, further research is needed to explore its structure, function, mechanisms of action, and immunomodulatory effects. By addressing current challenges through ongoing, in-depth studies, we anticipate the development of more effective cancer therapies targeting UBE2T.

## Figures and Tables

**Table 1:** Association of UBE2T with E3 Ligases or other interacting proteins in different cancers.

**Cancer Type**	**E3 Ligase/Interacting Protein**	**Biological Outcome**	**Reference**
GBM	RPL6 GRP78	Degrading RPL6 to suppress apoptosis and promoting proliferation; stabilizing GRP78 via Lys63-linked ubiquitination, enhancing EMT, invasion, and migration	[14,68]
HCC	Mule	Degrading Mule thus activating Wnt/β-catenin signaling and enhancing proliferation and stemness	[45]
PC	RING155	Degrading p53, reducing replication stress, enhancing pyrimidine biosynthesis and EMT	[62]
OC	p53	Promoting p53 ubiquitination and degradation, enhancing platinum resistance.	[69]
LUAD	PBX1 SORBS3	Ubiquitinating and degrading PBX1, negatively regulating RORA to promote proliferation and migration; degrading SORBS3 to activate IL-6/STAT3 signaling, driving tumor growth	[70,71]
GC	RACK1	Degrading RACK1, overactivating Wnt/β-catenin to drive tumor growth	[48]

## Data Availability

No new data were created or analyzed in this study.
